# Is the hypothesis of preimplantation genetic screening (PGS) still supportable? A review

**DOI:** 10.1186/s13048-017-0318-3

**Published:** 2017-03-27

**Authors:** Norbert Gleicher, Raoul Orvieto

**Affiliations:** 1 The Center for Human Reproduction, New York, NY 10021 USA; 2 Foundation for Reproductive Medicine, New York, NY 10022 USA; 3 Laboratory of Stem Cell Biology and Molecular Embryology, The Rockefeller University, New York, NY 10065 USA; 4 Department of Obstetrics and Gynecology, University of Vienna School of Medicine, 1090 Vienna, Austria; 5 Infertility and IVF Unit, Department of Obstetrics and Gynecology, Sheba Medical Center (Tel Hashomer), Sackler Faculty of Medicine, Tel Aviv University, Tel Aviv, Israel

**Keywords:** PGS, IVF, Mosacism, Blastocyst, Pregnancy rate, Live birth rate, CGH, NGS

## Abstract

The hypothesis of preimplantation genetic diagnosis (PGS) was first proposed 20 years ago, suggesting that elimination of aneuploid embryos prior to transfer will improve implantation rates of remaining embryos during in vitro fertilization (IVF), increase pregnancy and live birth rates and reduce miscarriages. The aforementioned improved outcome was based on 5 essential assumptions: (i) Most IVF cycles fail because of aneuploid embryos. (ii) Their elimination prior to embryo transfer will improve IVF outcomes. (iii) A single trophectoderm biopsy (TEB) at blastocyst stage is representative of the whole TE. (iv) TE ploidy reliably represents the inner cell mass (ICM). (v) Ploidy does not change (i.e., self-correct) downstream from blastocyst stage. We aim to offer a review of the aforementioned assumptions and challenge the general hypothesis of PGS. We reviewed 455 publications, which as of January 20, 2017 were listed in PubMed under the search phrase < preimplantation genetic screening (PGS) for aneuploidy>. The literature review was performed by both authors who agreed on the final 55 references. Various reports over the last 18 months have raised significant questions not only about the basic clinical utility of PGS but the biological underpinnings of the hypothesis, the technical ability of a single trophectoderm (TE) biopsy to accurately assess an embryo’s ploidy, and suggested that PGS actually negatively affects IVF outcomes while not affecting miscarriage rates. Moreover, due to high rates of false positive diagnoses as a consequence of high mosaicism rates in TE, PGS leads to the discarding of large numbers of normal embryos with potential for normal euploid pregnancies if transferred rather than disposed of. We found all 5 basic assumptions underlying the hypothesis of PGS to be unsupported: (i) The association of embryo aneuploidy with IVF failure has to be reevaluated in view how much more common TE mosaicism is than has until recently been appreciated. (ii) Reliable elimination of presumed aneuploid embryos prior to embryo transfer appears unrealistic. (iii) Mathematical models demonstrate that a single TEB cannot provide reliable information about the whole TE. (iv) TE does not reliably reflect the ICM. (v) Embryos, likely, still have strong innate ability to self-correct downstream from blastocyst stage, with ICM doing so better than TE. The hypothesis of PGS, therefore, no longer appears supportable. With all 5 basic assumptions underlying the hypothesis of PGS demonstrated to have been mistaken, the hypothesis of PGS, itself, appears to be discredited. Clinical use of PGS for the purpose of IVF outcome improvements should, therefore, going forward be restricted to research studies.

## Background

Women's fecundity decreases gradually with increasing age, accompanied by significant increases in the rates of aneuploidy and spontaneous miscarriages [1]. These observations have led to the attractively logical hypothesis of preimplantation genetic screening (PGS), that the transfer of only euploid embryos should improve IVF outcomes, with older women considered as the best candidates. However, PGS, first proposed by Verlinsky and Kuliev in 1996 [2], is a still unproven hypothesis, based on five assumptions: (i) Most in vitro fertilization (IVF) cycles fail because of aneuploid embryos. (ii) Their elimination prior to embryo transfer will, therefore, improve IVF outcomes. (iii) A single trophectoderm biopsy (TEB) at blastocyst stage is representative of the whole TE. (iv) TE ploidy reliably represents the inner cell mass (ICM). (v) Ploidy does not change (i.e., self-correct) downstream from blastocyst stage.

In view of increasing doubts about the general hypothesis of PGS, we here offer a review of these assumptions, demonstrating that none actually holds up to scrutiny. The hypothesis of PGS, therefore, appears increasingly difficult to maintain.

## Methods

We performed this review based on a primary literature search of 455 publications, which as of January 20, 2017, were listed in PubMed under the search phrase < preimplantation genetic screening (PGS) for aneuploidy>. The references of these manuscripts were further reviewed when considered relevant to the subject. The literature review was performed by both authors who agreed on the final 55 references.

## Discussion

### A history of PGS

PGS pioneers Verlinsky and Kuliev published a first report in 1996 [2]. The hypothesis, thus, just celebrated its 20th anniversary, without so far fulfilling its promise of improved IVF outcomes and reduced miscarriage rates [3–11]. Yet, PGS, nevertheless, thrived, reaching considerable utilization in IVF cycles [12]. Verlinsky and Kuliev initially proposed PGS by polar body biopsy [13] but, because technically simpler, practice quickly moved to more invasive cleavage stage (day-3) embryo biopsies [14].

This method of PGS, going forward described as PGS 1.0, achieved widespread popularity, even though a number of clinical trials in Belgium were unable to demonstrate outcome benefits [15–17]. Only once Mastenbroek et al published in 2007 their now “infamous” clinical trial [18], did professional organizations, acknowledge the futility of PGS 1.0 [19–21]. They, however, overlooked that the Mastenbroek study not only demonstrated lack of efficacy in improving IVF outcomes but, actually, in older women (i.e. poor prognosis patients) demonstrated harm in form of lower pregnancy rates. Harm in older women was also suggested by reanalysis of previously noted Belgian studies [3]. Moreover, based on proponents of PGS declaring the study by Mastenbroek et al as “seriously flawed” [22], PGS laboratories continued promoting PGS 1.0, and clinicians continued to recommend the procedure to patients. Paradoxically, the clinical utilization of PGS 1.0 continued without demands for proof of efficacy.

Even proponents of PGS, however, understood that the PGS procedure required improvements.

Because the basic hypothesis of PGS was still undisputed, most emphasis concentrated on technical aspects of the procedure. It was widely assumed that better techniques and technologies would lead to final validation of PGS by demonstrating expected outcome improvements [22]. That the basic hypothesis of PGS may be at fault, and that the effectiveness of PGS may vary in different patient populations was widely dismissed. Denial of the latter, despite Mastenbroek’s study [18], was, indeed, remarkable, as older women were still considered best candidates for PGS [23].

Arrival of new diagnostic technologies with clearly improved accuracy of chromosomal assessments offered ample opportunity for technical improvements. Those not only were more accurate than PGS 1.0 but also permitted investigations of complete chromosome complements in place of limited chromosome panels. By moving embryo biopsies from single (or double) blastomere biopsy at cleavage stage (day-3) to trophectoderm (TE) biopsy (TEB) at blastocyst stage (days 5/6), first proposed in 1990 [24], more genetic materials could be obtained, presumably rendering PGS (going forward, referred to as PGS 2.0) even more accurate [25]. As a result, the utilization of PGS 1.0 declined and was quickly replaced by PGS 2.0, skipping the prerequisite mandatory testing of PGS 2.0's accuracy, precision and whether it is affected by TE mosaicism [22, 26–28].

### Clinical outcomes of PGS 2.0

PGS 2.0, applying different platforms of comparative chromosome screening to detect embryonic aneuploidy was, thus, almost exclusively predicated on allegedly more accurate diagnosis of embryonic aneuploidies. Several studies claimed improved clinical IVF outcomes following PGS 2.0, recently summarized in a meta-analysis [29]. Among 29 eligible articles only three prospective trials and eight observational studies met even minimal inclusion criteria. The authors concluded that only in patients with normal ovarian reserve (i.e. good prognosis patients) PGS 2.0 significantly improved clinical and sustained pregnancy rates. Of notice, older poor prognosis patients were primarily considered the best candidates for PGS, not good prognosis women!

These conclusions are even more misleading, since in good prognosis patients all methods of embryo selection further improve IVF outcomes in patients who, even without embryo selection, achieve excellent pregnancy outcomes. They, therefore, need outcome improvements the least among IVF patients. Embryo selection methods usually, however, do not benefit average prognosis patients and are outright harmful to poor prognosis patients [30, 31], as previously also noted in association with PGS 1.0 [18].

Those study results also have to be questioned on statistical grounds since studies in the metaanalysis that favored PGS 2.0 were biased, as they uniformly only reported IVF outcomes following embryo transfers in first fresh IVF cycles. The relevant outcome parameter should, however, be total reproductive potential of each *initiated* IVF cycle. Analyses, therefore, should include fresh plus subsequent frozen/thawed transfers with reference point cycle start (i.e., intent to treat), and not, as in all included studies happened, with reference point embryo transfer, since analyses with reference point embryo transfer exclude poor prognosis patients who fail to reach transfer [30].

This was recently well demonstrated by Kang et al, when reporting PGS 2.0 effects on IVF above age [32]. With reference embryo transfer, they found significant improvements in clinical pregnancy and live birth rates. With reference point cycle start, results, however, differed remarkably [33]: As already demonstrated by Mastenbroek et al with PGS 1.0 [18], here the authors reported significantly lower clinical pregnancy and live birth rates (21.5% and 19.9%) in comparison to non-PGS patients (49.5% and 39.8%). Similar results were also reported by Kushnir et al after reanalyzing U.S. national PGS outcome data, initially erroneously reported to demonstrate outcome advantages for PGS [34]. That not a single PGS 2.0 study in the literature, claiming IVF outcome improvements, relied on outcome analyses with reference point cycle start is, therefore, telling.

Lacking properly conducted prospective clinical trials, a theoretical model was recently published for PGS 2.0, relying on evidence- based data in the literature on blastulation and aneuploidy rates, the rate of mosaicism, technical errors and implantation/live birth rates of PGS and non-PGS cycles at cleavage and blastocyst stage. The model clearly demonstrated superiority of non-PGS over PGS cycles for cumulative live birth rates (ranges, 18.2 – 50.0% vs 7.6 - 12.6%) [35].

### Accuracy and precision of PGS 2.0

Starting in 2015, clinical utility of PGS 2.0 faced increasing scrutiny. Aside from above-noted corrected re-analyses of published studies, the literature started reporting cases where patients experienced spontaneous miscarriages after PGS, which upon chromosomal reassessment were found to be aneuploid, raising the specter of false-negative TEBs [36]. At the same time, concerns about false-positive TEBs arose in relative good prognosis patients who repeatedly underwent IVF cycles without ever reaching embryo transfers because all embryos were reported as aneuploid. Suspicion that such patients may discard false-positive embryos, erroneously labeled as aneuploid, led us [32, 37] and others [38] to transfer such embryos, resulting in surprisingly high normal live birth rates and so far, no miscarriages.

The rate of TE mosaicism in human embryos has, however, remained controversial, reported as high as 70 and 90% in cleavage- and blastocyst-stage embryos, respectively [39], but increasingly believed a normal physiological phenomenon [40]. Mitotic, rather than meiotic errors appear to represent the majority [41]. While Liu et al. reported that 69% of abnormal blastocysts from women of advanced age are mosaic for ICM and TE [42], Johnson et al. demonstrated that in younger women 20% of blastocysts are aneuploid, with a majority of the abnormal blastocysts presenting with only one or two structural chromosome abnormalities [43], suggesting even in young women a still critical level of mosaicism at blastocyst stage [39].

These studies questioned one of the most basic argument for the switch from PGS 1.0 to PGS 2.0, - reduction in false-negative and false-positive embryo biopsies due to lower mosaicism risk with TEBs [22]. The opposite, indeed, appears to be the case: Similar to cancer cells, blastomeres of early stage human embryos demonstrate increased expression of gene products involved in cell cycle progression, while lacking expression of cell cycle checkpoint genes. This, potentially, increases mitotic error rates, causing genetic instability. Stress from extended embryo culture to blastocyst stage may, therefore, contribute to increased mosaicism [44].

Further evidence for a non-precise diagnoses due to TE mosaicism came from studies of multiple TEB biopsies, demonstrating up to 50% divergence between biopsies of same embryos in same laboratories, and up to approximately 80% divergence between multiple biopsies in different laboratories [32, 37, 45]. A recently published study evaluated in eight embryos concordance of multiple TEBs and in four embryos concordance of TE and ICM biopsies. Discordant results (i.e., mosaicism) were observed in 3/8 embryo [46].

TE mosaicism may, thus, be present in at least half of all embryos. In addition, laboratory platforms used in assessing TEBs may offer different diagnostic sensitivities and specificities in detecting chromosomally abnormal cell lines, further discussed below in a review of recently released practice guidelines for PGS 2.0 by the *Preimplantation Genetic Diagnosis International Society (PGDIS)* [47].

### Can we improve PGS 2.0 accuracy and precision?

The aforementioned observations are not surprising, since both the TE and the ICM are products of different cell lineages [48], with the ICM giving rise to the fetus, while the TE becomes placenta. Even in normal euploid offspring, the placenta has frequently been known to be seeded with islands of aneuploid cells [49]. This observation, alone, should, therefore, have led to caution about how TEBs are interpreted.

Supporting more mosaicism in TE than ICM are also recent mouse data, which demonstrated more efficient self-correction that eliminates aneuploid cell lineages in the ICM than in TE. The same mouse study also demonstrated considerable self-correction of even significant degrees of aneuploidy in the ICM downstream from blastocyst stage, resulting in 100% chromosomally normal pubs with up to half of ICM cells being aneuploid at blastocyst stage. Even with two-thirds of ICM cells aneuploid, a significant minority of pubs were chromosomally normal at birth [40]. If abnormal embryos at blastocyst stage still have the ability to self-correct downstream, assuming similar abilities in mouse and human embryos, any rational for blastocyst stage TEBs disappears.

Then the question arose whether a single TEB even can reliably define ploidy of the whole TE? Mathematical models, assuming a 6-cell TEB (the average reported cell number of a TEB) and a ca. 300-cell total TE [50], demonstrated that the likelihood of false-negative and false-positive diagnoses was too high to permit determination whether an embryo could be transferred or should be discarded [51]. The study, indeed suggested that a TEB would have to contain at least 27 cells to reach mathematical probability of accuracy. A higher cell number in a TEB might, therefore, increase PGS 2.0's precision. A recent study by study by Neal et al [52], however, invalidated this suggestion. These authors clearly demonstrated that the lowest live birth rates after single embryo transfer were associated with TEB with highest relative DNA content (high cell number), probably resulting from the mechanical impact of the biopsy.

Despite many obvious reasons to question the utility of PGS 2.0 in clinical practice, proponents of PGS are still advocating continuous clinical utilization of PGS [53]. Reemphasizing that the onus of validating the clinical effectiveness of PGS lies with proponents of the procedure appears, therefore, of importance. Also, effectiveness of clinical interventions should not be defined by non-inferiority, as has been suggested by some proponents of PGS 2.0 [54], but by statistical superior outcomes. In absence of superior clinical outcomes, additional costs and risks of a procedure like PGS do not appear warranted.

The most recent published opinion on "detecting mosaicism in trophectoderm biopsies" pointed out that even PGS 2.0 still demonstrates significant technical shortcomings [53] but, once again, only offered mostly technical explanations why neither PGS 1.0 nor PGS 2.0 ever was able to reliably determine whether embryos are chromosomally normal or not (i.e., transferrable or not).

### Guidelines from professional societies

Despite so much emphasis on procedural and technical aspects of PGS, remarkably, neither professional societies, like the *American Society for Reproductive Medicine (ASRM)* or the *European Society for Human Reproduction and Embryology (ESHRE)* have in respect to PGS 2.0 commented on either. Neither have national authorities, like the Food and Drug Administration (FDA) in the U.S. or the Human Fertilization and Embryology Authority (HFEA) in the U.K., though the latter in a recent BBC broadcast announced a possible review during 2017.

The only society, which commented on the utilization of PGS 2.0 following above outlined recent developments and the recognition that TE mosaicism at blastocyst stage is significantly more prevalent than previously assumed, has been the *PGDIS*, which recently issued radically revised new guidelines on how PGS 2.0 should be performed, laboratory reports should be issued and how clinicians should interpret these reports [50]. They are here reprinted as Tables 1, 2 and 3.

**Table 1:** *PGDIS* Recommendations for PGS laboratories [47]

1	For reliable detection of mosaicism, ideally 5 cells should be biopsied, with as little cell damage as possible. If the biopsy is facilitated using a laser, the identified contact points should be minimal and preferably at cell junctions. Overly aggressive use of the laser may result in cell damage and partial destruction of cellular DNA.
2	Only a validated Next Generation Sequencing (NGS) platform that can quantitatively measure copy number should be used for measurement of mosaicism in the biopsy sample. Ideally, a NGS methodology that can accurately and reproducibly measure 20% mosaicism in a known sample.
3	For reporting embryo results, the suggested cut-off point for definition of mosaicism is >20%, so lower levels should be treated as normal (euploid), > 80% abnormal (aneuploid), and the remaining ones between 20-80% mosaic (euploid-aneuploid mosaics).

**Table 2:** *PGDIS* recommendations for the clinician [47]

1	Patients should continue to be advised that any genetic test based on sampling one or small number of cells biopsied from preimplantation embryos cannot be 100% accurate for a combination of technical and biological factors, including chromosome mosaicism.
2	The patient information and consent forms for aneuploidy testing (if used) should be modified to include the possibility of mosaic aneuploid results and any potential risks in the event of transfer and implantation. This needs to be explained to patients by the clinician recommending the aneuploidy testing.
3	Transfer of blastocysts with a normal euploid result should always be prioritized over those with mosaic aneuploid results.
4	In the event of considering the transfer of a blastocyst with only mosaic aneuploidies, the following options should be discussed with the patient:
	a. A further cycle of IVF with aneuploidy testing to increase the chance of identifying a normal euploid blastocyst for transfer
	b. Transfer of a blastocyst with mosaic aneuploidies for low risk chromosomes only, after appropriate genetic counseling if available
	c. Appropriate monitoring and prenatal diagnosis of any resulting pregnancy, preferably by early amniocentesis (>14 weeks gestation)

**Table 3:** *PGDIS* guidelines to prioritize mosaic embryos for transfer [47]

Based on our current knowledge of the reproductive outcomes of fetal and placental mosaicism from prenatal diagnosis, the following can be used as a guide by the clinician (or a genetic counselor if available) when a mosaic embryo is being considered for transfer:	
1	Embryos showing mosaic euploid/monosomy or mosaic euploid/ monosomy are preferable to euploid/trisomy, given that monosomic embryos (excepting 45, X) are not viable
2	If a decision is made to transfer mosaic embryos trisomic for a single chromosome, one can prioritize selection based on the level of mosaicism and the specific chromosome involved
	a. The preferable transfer category consists of mosaic embryos trisomic for chromosomes 1, 3, 4, 5, 6, 8, 9, 10, 11, 12, 17, 19, 20, 22, X, Y. None of these chromosomes involve the adverse characteristics enumerated below.
	b. Embryos mosaic for trisomies that are associated with potential for uniparental disomy [14, 15] are of lesser priority
	c. Embryos mosaic for trisomies that are associated with intrauterine growth retardation (chromosomes 2, 7, 16) are of lesser priority
	d. Embryos mosaic for trisomies capable of liveborn viability (chromosomes 13, 18, 21) are for obvious reasons of lowest priority

In these guidelines the Society acknowledged that technical abilities of PGS 1.0 and PGS 2.0 have been inadequate and, therefore, established new guidelines for diagnostic platforms to be used, defined new diagnostic definitions for embryos and greatly expanded on which embryos potentially could be transferred. It, thereby, implicitly acknowledged that large numbers of, likely, normal-mosaic embryos had been erroneously discarded in the past.

Since until recently PGS laboratories recommended the discarding of any embryo in presence of any observed aneuploidy, the radical nature of these revised guidelines cannot be overemphasized. We, therefore, will address them in some detail.

In recommendations to PGS laboratories (Table 1), the *PGDIS* advised that, since only Next Generation Sequencing (NGS) is capable of measuring chromosomal copy numbers, NGS should be the only diagnostic platform used in assessing TE mosaicism in association with PGS 2.0. The society, however, also acknowledged, that even NGS is only able to detect mosaicism above a 20% threshold. T, therefore, changed the definition of a “normal (euploid)” embryos to TE mosaicism below 20% (Table 1). Even “normal” embryos, therefore, now may be up to 20% TE-mosaic.

Since most published TE mosaicism rates in the literature were based on other than NGS platforms and, since even NGS does not detect *all* TE mosaicism, the *PGDIS*, therefore, explicitly acknowledged that the literature so far has significantly underestimated the true rate of TE mosaicism.

TE biopsies 20-80% mosaic, under these new *PGDIS* guidelines, are defined as “euploid-aneuploid mosaic”, (Table 1), and are potentially transferrable (Table 2). Only biopsies above 80% mosaic are now considered truly “aneuploid” (Table 1), and to be discarded.

Considering that until very recently embryos with even most minute degrees of aneuploidy were considered “aneuploid” and, therefore, discarded, these new diagnostic criteria are truly groundbreaking. They, however, unfortunately, once again are completely arbitrary, and lack any evidence-based support. No data exist in the literature to validate the *PGDIS’* new cut-off ranges that define above described new diagnostic criteria for embryos. They, therefore, still, do not permit reliable determinations whether biopsied embryos should be transferred or discarded.

To a degree this is acknowledged by the PGDIS in their recommendations to clinicians (Table 2), which for the first time offer the option of embryo transfers with up to 80% mosaic TEBs, and in Table 3, indeed, offer a hierarchy for such transfers.

These recommendations are, however, as of this point uniformly speculative. Some recommendations even are counterintuitive, like the recommendation that the first consideration if all embryos in an IVF cycle are reported to be “euploid-aneuploid mosaic,” should be *“a further cycle of IVF with aneuploidy testing to increase the chance of identifying normal euploid blastocysts to transfer.”* Why under such circumstances a patient with transferrable “euploid-aneuploid mosaic” embryos should undergo additional treatments, and face additional costs for another fresh IVF cycle, appears unclear.

Most remarkable about these revised guidelines is, however, the complete absence of any discussion about the clinical utility of PGS 2.0. Considering all here outlined shortcomings of PGS 2.0, implicitly acknowledged by the *PGDIS* in these new guidelines, one is left wondering why PGS 2.0 should still be applied?

## Conclusions

We started this review with the statement that, based on five assumptions, PGS has remained an unproven hypothesis. We have here demonstrated that all five of these assumptions, indeed, are incorrect: (i) The long-held association of embryo aneuploidy with IVF failure has to be reevaluated, and published statistical associations have to be reconsidered in view how common TE mosaicism is. (ii) Reliable elimination of presumed aneuploid embryos prior to embryo transfer appears unrealistic. (iii) Mathematical models demonstrate that a single TEB cannot provide reliable information about the chromosomal constituency of the whole TE, and larger biopsies reduce blastocysts' implantation potential. (iv) TE, as site of every embryo biopsy in PGS 2.0, does not reliably reflect the ICM. And (v) embryos, with great likelihood, still have strong innate abilities to self-correct downstream of blastocyst stage, with ICM doing so better than TE.

Considering that we, in addition, also demonstrated that not one properly analyzed study has been able to demonstrate clinical outcome benefits for PGS 2.0 and, indeed, increasing evidence suggests that PGS 2.0, at least in unfavorable patient populations, who were considered best candidates for PGS 2.0, may actually reduce pregnancy and live birth chances, it is difficult to understand why PGS 2.0 should still be performed in association with IVF. The only potential justification for the utilization of PGS 2.0, based on current knowledge is in our opinion in good prognosis patients with large numbers of high quality of embryos who have failed multiple prior embryo transfers.

Short of interventions by the FDA in the U.S. or by other regulatory agencies elsewhere, going forward, the responsibility for protecting patients from exaggerated claims about PGS 2.0 lies with providers of clinical IVF services who, ultimately, decide whether their patients should be exposed to PGS or not. Based on here presented data, we can only encourage clinicians to concentrate on best interests of their patients. The PGS laboratory community appears determined to continue to build expectations that yet another “better” PGS will come along [55]. The introduction of PGS 3.0, therefore, may be just around the corner!

## Abbreviations

ASRM: American society for reproductive medicine
ESHRE: European society for human reproduction and embryology
FDA: Food and drug administration
HFEA: Human fertilization and embryology authority
ICM: Inner cell mass
IVF: In vitro fertilization
NGS: Next generation sequencing
PGDIS: Preimplantation genetic diagnosis international society
PGS: Preimplantation genetic diagnosis
TE: Trophectoderm
TEB: Trophectoderm biopsy
